# Attitudes Toward Large Language Models in Health Care and Preferences for Their Adoption and Oversight Among Health Care Professionals: Cross-Sectional Survey

**DOI:** 10.2196/87818

**Published:** 2026-09-15

**Authors:** Arya Rao, Chinemerem Nwokemodo-Ihejirika, John W R Kincaid, Marharyta Krylova, Kaiz P Esmail, Dan Nguyen, Christian Rivera, Erica Koranteng, Marc D Succi

**Affiliations:** 1 Harvard Medical School, Boston, MA, United States; 2 Succi Lab, Mass General Brigham, Boston, MA, United States; 3 Department of Radiology, Mass General Brigham, Boston, MA, United States; 4 University of Massachusetts Chan Medical School, Worcester, MA, United States

**Keywords:** large language models, clinician attitudes, clinical decision support, technology adoption, AI implementation barriers, AI in health care

## Abstract

**Background:**

Large language models (LLMs) are rapidly entering health care, but limited empirical data exist on health care professionals’ perceptions. Understanding health care professionals’ attitudes is essential for responsible implementation as LLMs transition from experimental to routine tools.

**Objective:**

To characterize health care professionals’ perspectives on LLM use in health care, including exposure, knowledge, perceived clinical utility, safety and bias concerns, and oversight preferences.

**Methods:**

This cross-sectional survey was distributed online through a health care news platform mailing list. A total of 335 health care professionals responded, including attending physicians (n=230, 68.7%), residents or fellows, nurse practitioners, physician assistants, and researchers. Most were aged 30 to 59 years (n=243, 72.5%) and practiced in the Northeast United States (n=261, 77.9%). Outcomes included LLM use patterns, knowledge levels, perceived applications, safety and bias concerns, and preferences for regulatory oversight. Analyses included descriptive statistics, Wilcoxon rank-sum tests, *χ*² tests, and Spearman correlations.

**Results:**

Of 335 participants, 62.7% (n=210) reported current or contemplated LLM use. Users reported significantly higher self-reported knowledge than nonusers (*P*<.001). Age was not associated with knowledge (ρ=–0.072; *P*=.19). Participants identified literature review (n=246, 73.4%), decision support (n=191, 57%), and patient communication (n=184, 54.9%) as the most valuable applications. Concerns included decision errors (n=253, 75.5%) and algorithmic bias (n=245, 73.1%); nearly all respondents (n=323, 96.4%) expressed concern about bias, and those who had observed bias reported higher concern levels (*P*<.001). Participants favored regulation by professional associations (n=219, 65.4%) over technology companies (n=97, 29%), with 87.8% (n=294) supporting professional guidelines. Confidence in existing oversight was low, with 66.6% (n=223) reporting none.

**Conclusions:**

In this exploratory convenience sample, health care professionals reported early adoption of LLMs for lower-risk tasks while expressing concerns about safety, bias, and governance. Given the low response rate and recruitment through a health care innovation–focused mailing list, these findings may not reflect the views of the broader health care professional population. Respondents preferred professional organizations over industry for oversight and suggested that successful integration of LLMs into health care will require careful planning, human supervision, transparent disclosure, and auditing. Future studies using more representative sampling methods are needed to better characterize health care professionals’ attitudes toward LLMs.

## Introduction

Large language models (LLMs) have rapidly entered the health care landscape, offering new capabilities across a range of clinical and operational domains [1], including diagnosis and treatment [2-11], patient communication [12-18], clinical documentation [19-25], and medical education [26-29]. Trained on vast corpora of text, these models use deep learning to generate human-like responses and perform a wide range of cognitive tasks. Alongside this expanding scope of application, physician adoption of AI tools has accelerated markedly, with reported use rising from 38% in 2023 to 66% in 2024 [30].

Despite this rapid adoption, LLMs carry important limitations that are particularly consequential in high-stakes clinical environments. Most prominently, these models are susceptible to generating false or hallucinated information [31], a phenomenon documented across specialties and model architectures, with rates that vary considerably by task complexity and knowledge domain. Ethical concerns also remain underrecognized, including the inherent biases of machine learning, a lack of transparency in the composition of training datasets, and underdeveloped regulatory frameworks [32]. Algorithmic bias in health AI can emerge at multiple stages of model development, including training datasets, feature selection, and model architecture; these biases have been shown to propagate and amplify preexisting disparities in health care access and outcomes [33,34]. Compounding these concerns, the regulatory landscape for LLMs in health care remains fragmented; standardized predeployment validation requirements are lacking, and current oversight mechanisms are poorly suited to the iterative update cycles that characterize modern LLM development [35,36]. These conditions may leave health care professionals to navigate significant uncertainty when adopting or overseeing these tools in clinical workflows.

Literature examining health care professionals’ perceptions of these tools has begun to emerge [1-7,9,10,12-16,19,26-29,37,38]. Early systematic reviews synthesized health care professional and public attitudes toward clinical AI broadly, but predated the widespread availability of conversational LLMs, and did not examine LLM-specific concerns or governance priorities [39]. Specialty-specific surveys have similarly found that health care professionals broadly recognize AI’s potential, yet express concerns about accuracy and reliability, and the adequacy of training [40,41].

Two recent surveys have specifically examined LLM perceptions among health care professionals. Ozkan et al [42] conducted a cross-sectional online survey, finding that 76.2% of health care professionals across 21 countries had used ChatGPT in manuscript writing, clinical question–answering, and patient communication. Sumner et al [43] surveyed more than 800 health care workers, support staff, students, and academics, finding that 75% of LLM users reported a positive experience overall, though 46% found generated content unhelpful. Together, these studies establish that LLM adoption among health professionals is underway and concerns are widespread. However, both studies were international rather than US-focused, and neither examined preferences for regulatory oversight, institutional governance structures, or confidence in existing regulatory frameworks—dimensions that are increasingly critical as LLMs transition from experimental to routine clinical use.

This study addresses these gaps by providing insights into LLM-specific perceptions across a multidisciplinary sample of US health care professionals. We capture preferences for oversight and regulation—including the degree of confidence health care professionals place in existing frameworks—and examine whether firsthand observation of biased LLM outputs predicts higher concern levels.

## Methods

### Study Design

This was a cross-sectional survey study, designed to characterize attitudes, knowledge, and current practices pertaining to LLM use among health care professionals. The study adhered to STROBE (Strengthening the Reporting of Observational Studies in Epidemiology) reporting guidelines for observational studies.

### Setting and Recruitment

The survey was distributed via the MESH Incubator mailing list, a listserv maintained by our institutional innovation center comprising health care professionals, researchers, and trainees who opted in via our online website [44], including both internal Mass General Brigman affiliates and external subscribers across the United States. This email listserv is comprised of health care providers who subscribed to the MESH Incubator newsletter and email update list or attended a prior health care education program from the MESH Incubator. No formal eligibility criteria restricted survey receipt beyond active subscription to this list. No restriction to prevent duplicate responses was applied in the REDCap survey. The survey was open for data collection from January 3 to February 7, 2025. Participation was voluntary and nonincentivized.

### Participant Eligibility

Eligible participants included any health care professional who received the survey invitation and submitted a fully completed response. There were no minimum experience requirements or specialty restrictions. Only fully completed responses were included in the analysis to preserve the consistency and rigor of the dataset. There were no partially completed responses in the final sample.

### Survey Development

We developed a survey to operationalize 5 core constructs: (1) LLM exposure and patterns of use, (2) self-reported knowledge, (3) perceived areas of clinical utility, (4) safety and bias concerns, and (5) preferences for regulatory oversight. Face validity was established through multiround review by the multidisciplinary authorship team. Items were revised iteratively until consensus on face validity, construct clarity, and response-option completeness was achieved. The final instrument was semistructured and composed of a combination of multiple-choice, Likert-type, and open-ended questions (see Multimedia Appendix 1 for full survey). The survey was not formally pilot-tested prior to deployment. No additional psychometric validation was performed prior to administration. The reliability, construct validity, and measurement properties of the instrument are unknown.

### Survey Measures

LLM use was assessed via a binary yes/no question. General opinion toward LLM use was captured on a 6-point scale, with an additional “N/A – have not used an LLM” response option to distinguish nonusers from those with negative attitudes. LLM knowledge was assessed on a 5-point ordinal scale (1, “not at all knowledgeable” to 5, “extremely knowledgeable”). Perceived application areas were assessed via multiselect “all that apply” items. Concern about bias in LLM development and confidence in current regulatory efforts were each assessed on 5-point ordinal scales (1, “not at all knowledgeable” to 5, “extremely knowledgeable”). Specific safety concerns, mechanisms of potential health inequities, and regulatory preferences were assessed via multiselect “all that apply” items. Demographic characteristics collected included professional role, age range, years in practice (for attending physicians), medical specialty (for attending physicians), and geographic regions of practice.

### Data Collection

Survey answers and all quantitative data were collected and analyzed in REDCap [45]. Of 22,918 individuals who received the survey invitation, 335 submitted fully completed responses, yielding a response rate of 1.46%. This response rate reflects the voluntary, nonincentivized nature of participation. Although the survey platform did not restrict multiple submissions, each respondent was assigned a system-generated participant ID. Prior to analysis, participant IDs and survey completion status were reviewed to identify duplicate or incomplete responses. All participant IDs were unique, and all responses were marked as complete; therefore, no responses were excluded as duplicates or incomplete. Respondents could also optionally provide an email address. However, the dataset was fully anonymized before analysis. The final analytic sample included all eligible unique respondents who completed the survey during the study period. The study population represents a self-selected convenience sample of health care professionals.

### Statistical Analysis

All statistical analyses were conducted using R (version 4.5.2; R Foundation for Statistical Computing). Descriptive statistics were calculated for demographic characteristics and survey responses; categorical variables are presented as frequencies and percentages.

Statistical tests were selected according to the measurement level of each variable and the nature of the comparison being made. Differences in self-reported LLM knowledge scores between LLM users and nonusers were assessed using the Wilcoxon rank sum test, appropriate for comparing an ordinal outcome between 2 independent groups. The relationship between respondent age and self-reported LLM knowledge was examined using Spearman rank correlation coefficient, as both variables were ordinal.

Differences in concern levels between respondents who had personally observed biased LLM outputs and those who had not, or were unsure, were assessed using both a *χ*² test of independence, treating concern level as categorical, and a Kruskal-Wallis H test, treating concern level as an ordered categorical variable to account for its ordinal structure. Cross-tabulation analysis was used to descriptively examine whether preferred safeguards against LLM-mediated inequity varied across primary concern categories.

The relationship between bias concern level and confidence in current LLM regulation was examined using the Spearman rank correlation coefficient. The relationships between the total number of concerns endorsed (concern count) and support for specialized training requirements, and separately between concern count and confidence in current regulatory oversight, were each examined using the Spearman rank correlation coefficient. Associations between bias concern level and support for mandatory disclosure requirements, and separately between bias concern level and support for specialized training requirements, were assessed using *χ*² tests of independence.

Only fully completed survey responses were included in analysis, so no imputation for missing data was required. Given the exploratory nature of this study and the relatively small sample size for subgroup analyses, no correction for multiple comparisons was applied. No multivariable or adjusted analyses were planned or performed; all reported associations are unadjusted bivariate comparisons. Statistical significance was set at α=.05 for all analyses.

### Ethical Considerations

This study was reviewed and deemed exempt by our institution’s institutional review board (2024P000379). All participants provided implied consent prior to initiating the survey. Survey responses were collected via REDCap. Respondents could optionally provide an email address; however, the analytic dataset was anonymized and contained no personal identifiers. Data were stored in a secure, access-controlled REDCap environment in compliance with institutional data governance policies.

## Results

### Study Demographics

A total of 335 health care professionals participated in the survey from January 3 to February 7, 2025 (Table 1). The majority of respondents were attending physicians (n=230, 68.7%), followed by research personnel (n=52, 15.5%), and other health care roles (eg, medical physicist, clinical psychologist; n=33, 9.9%), with smaller representation from residents or fellows (n=9, 2.7%), medical or physician assistant students (n=5, 1.5%), and advanced practice providers (n=6, 1.8%). Most were aged 30‐59 years (n=243, 72.5%) and practiced in the Northeast (n=261, 77.9%). Among attending physicians, mean years in practice was 19.6 (SD 12.4; range 1‐53). Internal medicine (n=53, 23%) and psychiatry (n=25, 10.9%) were the most represented specialties.

**Table 1.** Demographic and professional characteristics of 335 health care professionals and research personnel participating in a cross-sectional online survey on health care professionals’ perspectives toward LLM use in health care, distributed via a health care news platform mailing list in the United States.

Characteristic		Respondents, n (%)^a^
Professional role		
	Attending physician	230 (68.7)
	Research personnel	52 (15.5)
	Resident or fellow physician	9 (2.7)
	Medical/physician assistant student	5 (1.5)
	Mid-level health professional (nurse, physician assistant, nurse practitioner)	6 (1.8)
	Other^b^	33 (9.9)
Age group (years)		
	18‐29	13 (3.9)
	30‐44	131 (39.1)
	45‐59	112 (33.4)
	60‐64	31 (9.3)
	≥65	46 (13.7)
	Not available	2 (0.6)
Geographic region		
	Northeast	261 (77.9)
	Midwest	23 (6.9)
	West	21 (6.3)
	Southeast	19 (5.7)
	Southwest	11 (3.3)
Medical specialty^c^		
	Internal medicine	53 (23.0)
	Surgery (general, plastic, cardiothoracic, neurosurgery, urology)	12 (5.2)
	Psychiatry	25 (10.9)
	Pediatrics	13 (5.7)
	Anesthesia	10 (4.3)
	Obstetrics/gynecology	10 (4.3)
	Emergency medicine	9 (3.9)
	Radiology	6 (2.6)
	Cardiology	6 (2.6)
	Infectious diseases	5 (2.2)
	Dermatology	3 (1.3)
	Gastroenterology	3 (1.3)
	Hematology/oncology	3 (1.3)
	Endocrinology	3 (1.3)
	Nephrology	1 (0.4)
	Other specialties^d^	68 (29.6)

a Percentages may not sum to 100% due to rounding.

b Other roles include medical physicist, clinical psychologist, doctor of pharmacology, health care executive, registered nurse, researcher, physical therapist, and medical scribe.

c Attending physicians only (n=230).

d Other specialities include radiation oncology, palliative care, neurocritical care, neurology, pathology, ophthalmology, otolaryngology, rheumatology, pulmonology, allergy/immunology, and more.

### LLM Adoption and Self-Reported Knowledge

**Figure 1.**
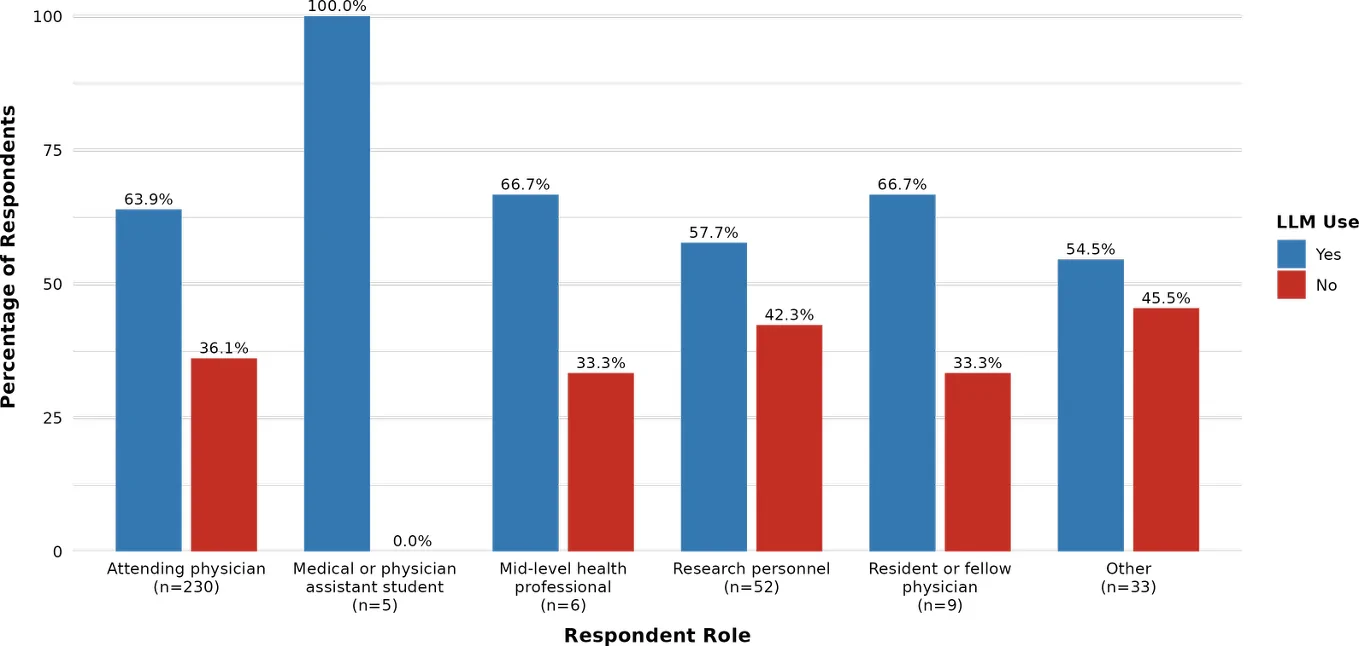
Large language model (LLM) use rates by health care professional role among 335 survey respondents. Bars represent the percentage of respondents within each role who reported using (blue) or not using (red) LLMs. Other roles include medical physicist, clinical psychologist, doctor of pharmacology, health care executive, registered nurse, researcher, physical therapist, and medical scribe.

**Figure 2.**
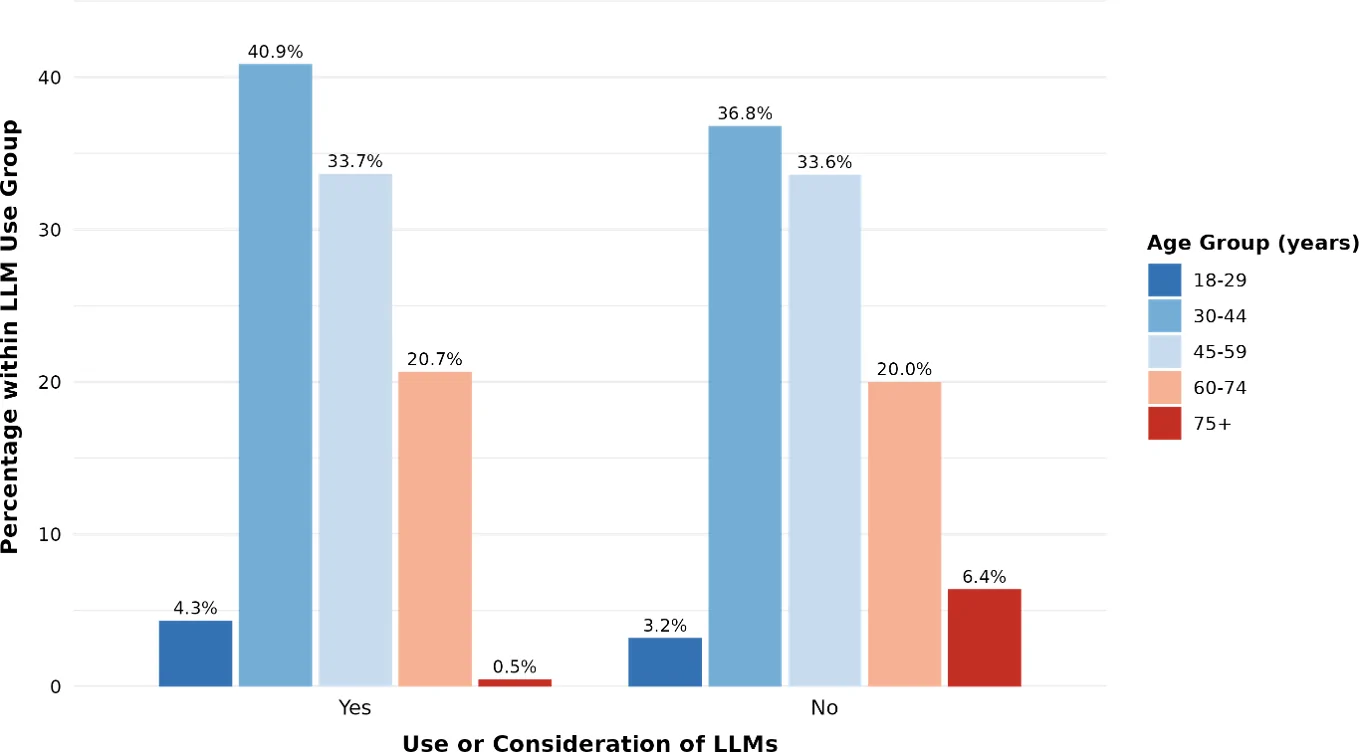
Age distribution of health care professionals by large language model (LLM) use status. Bars represent the percentage of respondents within each LLM use group (yes vs no) who fall into each age category (18‐29, 30‐44, 45‐59, 60‐74, and ≥75 years).

A majority of respondents (n=210, 62.7%) reported current or contemplated LLM use, with use distributed across all professional roles (Figure 1). Medical or physician assistant students showed the highest LLM use rate (n=5, 100% of this role), while research personnel showed the lowest (n=52, approximately 58% of this role). Age distribution was comparable between users and nonusers, both concentrated in the 30‐44 (n=85, 40.9%) and 45‐59 (n=70, 33.7%) age groups (Figure 2).

**Figure 3.**
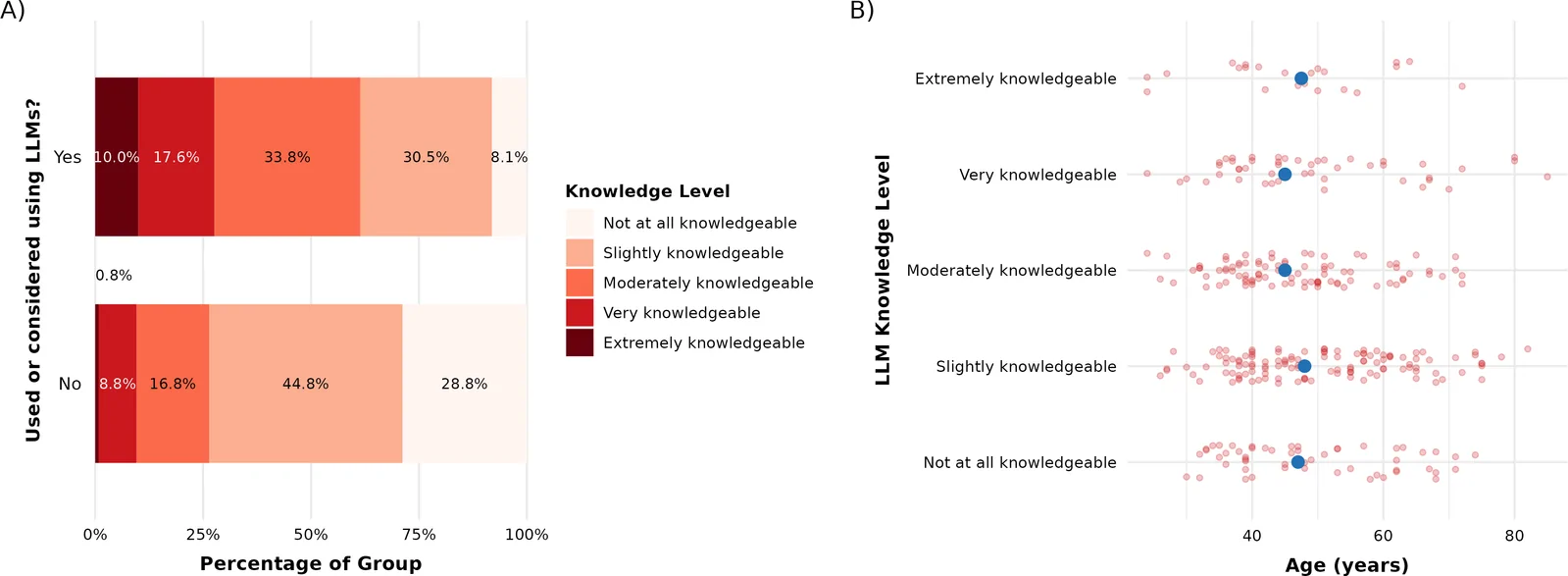
Self-reported large language model (LLM) knowledge levels, use status, and age among survey participants. (A) stacked horizontal bar chart showing the distribution of self-reported LLM knowledge (5 ordinal levels: “not at all” to “extremely knowledgeable”) among the respondents who reported current or contemplated LLM use (yes; n=210) versus those who did not (no; n=125); (B) strip plot showing self-reported LLM knowledge level plotted against respondent age; blue filled circles represent group median ages at each knowledge level.

LLM users reported markedly higher self-reported knowledge than nonusers; 27.6% (n=58) of users rated themselves as very or extremely knowledgeable, compared with 9.6% (n=12) of nonusers (Wilcoxon rank sum test: W=7571.5; *P*<.001; Figure 3A). In contrast, age was not a significant predictor of LLM knowledge (ρ=–0.072; *P*=.19; Figure 3B), suggesting that generational differences did not drive self-reported proficiency in this sample.

### Perceived Clinical Applications

Respondents most frequently identified researching medical literature as the highest-value LLM application (n=246, 73.4%), followed by clinical decision support (n=191, 57%), and answering patient questions (n=184, 54.9%). Insurance approvals (n=178, 53.1%), billing (n=164, 49%), and patient health monitoring (n=115, 34.3%) were endorsed less frequently (Table 2). An additional 16.4% (n=55) suggested applications beyond those listed.

**Table 2.** Perceived areas of greatest utility for large language models (LLMs) in health care practice reported by 335 health care professionals. Respondents could select multiple options.

Area of application	Respondents (N=335), n (%)
Clinical decision support	191 (57.0)
Answering patient questions	184 (54.9)
Researching medical literature	246 (73.4)
Monitoring patient health	115 (34.3)
Insurance approvals	178 (53.1)
Billing	164 (49.0)
Other	55 (16.4)

### Safety and Bias Concerns

**Figure 4.**
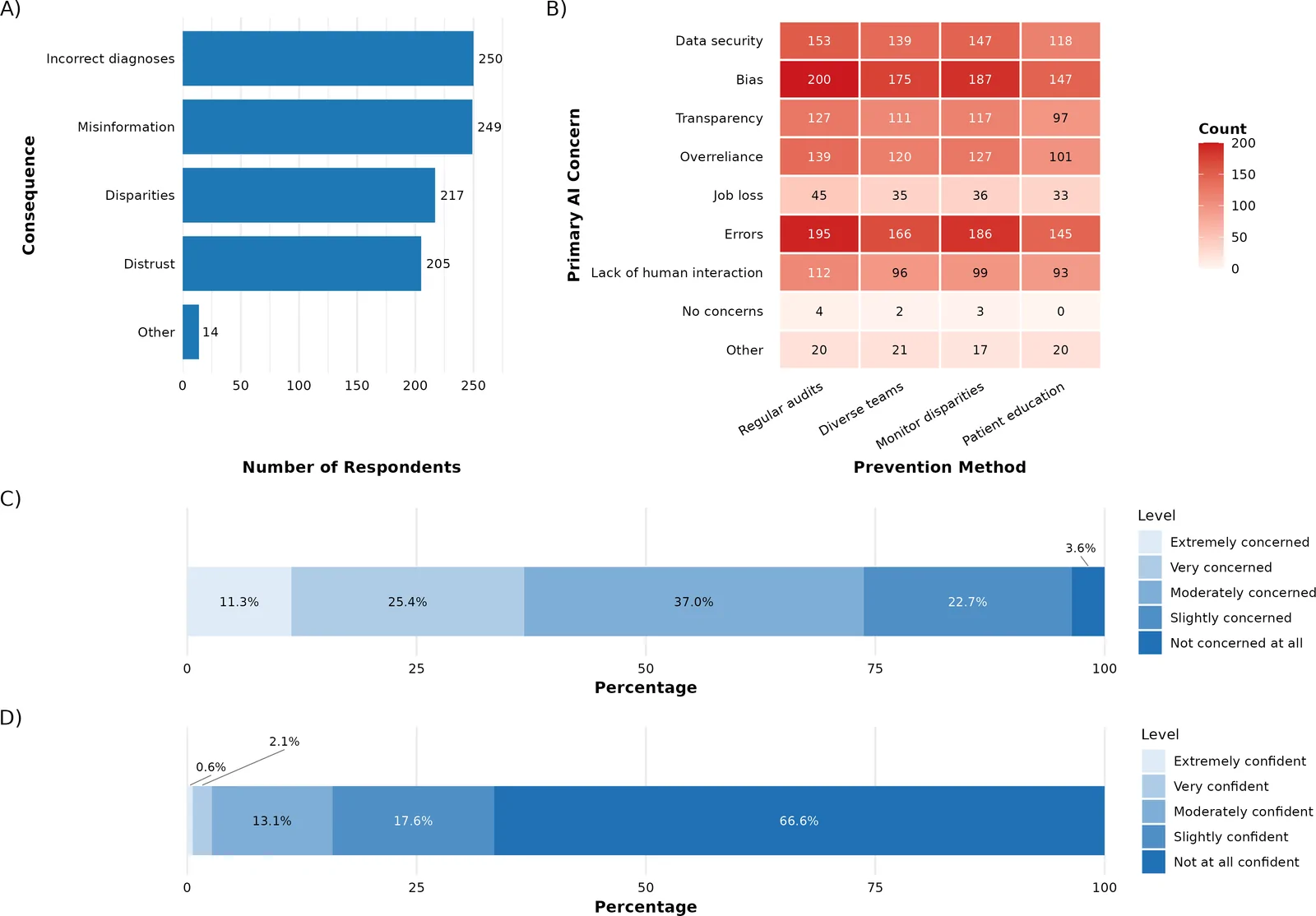
Safety concerns, bias prevention preferences, and regulatory confidence regarding large language model (LLM) use in health care. (A) Horizontal bar chart showing the number of respondents (out of 335; multiple selections permitted) endorsing each anticipated consequence of LLM bias in health care; (B) heatmap cross-tabulating respondents’ primary AI concerns (rows) against their preferred method for preventing AI-related injustice (columns); color intensity represents respondent count, with errors and bias showing the strongest endorsement of regular audits and disparity monitoring; (C) stacked horizontal bar chart displaying the distribution of concern levels about LLM bias across all respondents (5 ordinal levels: “not concerned at all” to “extremely concerned”); (D) stacked horizontal bar chart displaying respondent confidence levels in current AI regulatory frameworks (5 ordinal levels: “not at all confident” to “extremely confident”).

Concern about LLM implementation was near universal, with only 1.5% of 335 respondents (n=5) reporting no concerns. The most prevalent concerns were errors in decision-making (n=253, 75.5%) and algorithmic bias (n=245, 73.1%), followed by data security and privacy (n=187, 55.8%), overreliance on technology (n=172, 51.3%), and lack of transparency in decision-making (n=153, 45.7%; Figure 4B).

Concern about bias was high; 96.4% (n=323) of respondents expressed at least some degree of concern, with most falling in the moderately to very concerned range (Figure 4C). Among respondents who had personally observed biased LLM outputs (n=53, 15.8%), concern levels were significantly higher than among those who had not observed bias or were unsure (*χ*²_8_=56.92; *P*<.001; Kruskal-Wallis H=42.95; *P*<.001). This subgroup also demonstrated heightened concern across all anticipated consequences of bias, including incorrect diagnoses (n=44, 83%), patient misinformation (n=42, 79.2%), health care disparities (n=36, 67.9%), and erosion of professional trust (n=33, 62.3%), compared to the overall sample, which most commonly cited misinformation (n=249, 74.3%) and diagnostic inaccuracies (n=250, 74.6%) as the primary harms. Bias in LLM decision-making was the most frequently cited mechanism by which LLMs could perpetuate inequity (n=251, 74.9%), followed by privacy and security risks (n=145, 43.3%), and differential health care access (n=138, 41.2%).

Regardless of specific concern type, respondents consistently endorsed regular audits and assessments and monitoring of health care disparities as preferred safeguards against LLM-mediated inequity, a pattern that held across the primary concern categories examined in cross-tabulation analysis (Figure 4B). Among respondents who cited errors in decision-making as a concern, 195/253 (77.1%) endorsed regular audits and 186/253 (73.5%) endorsed disparity monitoring; among those concerned about algorithmic bias, 200/245 (81.6%) and 187/245 (76.3%); among those concerned about data security and privacy, 153/187 (81.8%) and 147/187 (78.6%); among those concerned about overreliance on technology, 139/172 (80.8%) and 127/172 (73.8%); and among those concerned about lack of transparency in decision-making, 127/153 (83.0%) and 117/153 (76.5%), respectively.

### Regulatory Preferences and Oversight Confidence

Respondents most commonly identified professional medical associations as the appropriate regulatory body (n=219, 65.4%), followed by health care organizations and hospitals (n=201, 60%) and government agencies (n=174, 51.9%); technology companies were supported by only 29% (n=97). Regarding regulatory mechanisms, 88% (n=295) endorsed guidelines and standards developed by professional organizations, 60.3% (n=202) supported government-enforced regulations, and only 20.3% (n=68) favored self-regulation by technology companies.

Confidence in current regulation was low, with 67% (n=224) of respondents reporting no confidence at all in existing LLM oversight (Figure 4D). Greater bias concern was significantly associated with lower regulatory confidence (ρ=−0.35; *P*<.001). Higher aggregate concern counts were positively correlated with support for specialized training requirements (ρ=0.145; *P*=.008) and inversely correlated with regulatory confidence (ρ=−0.185; *P*<.001), suggesting respondents experiencing broader concern about LLM risks were systematically more likely to favor structured accountability mechanisms and less likely to trust existing frameworks to provide them. This pattern was corroborated by significant associations between bias concern level and support for both mandatory disclosure requirements (*χ*²_8_=55.65; *P*<.001) and specialized training (*χ*²_8_=27.44; *P*<.001).

## Discussion

### Principal Findings

In this exploratory cross-sectional survey of 335 health care professionals and health professionals, we aimed to characterize the current adoption, perceived utility, and concerns pertaining to LLMs in clinical practice. Nearly two-thirds of respondents reported current or contemplated use of LLMs. Adoption spanned roles, specialties, and levels of training. Respondents most often identified literature review, clinical decision support, and patient communication as high-value applications. Notably, while use correlated with higher self-reported knowledge, age was not a meaningful predictor of LLM literacy or adoption.

Within this sample, respondents most commonly described LLM use in tasks that are lower-risk, high-leverage tasks that augment rather than replace clinical judgment [46]. In these domains, LLMs function as drafting aids, retrieval tools, and translators, with outputs subject to professional review prior to clinical use [47-49]. This alignment between the perceived utility of LLMs and their current restricted use cases indicates a pragmatic posture among health care professionals in this study; adoption of these tools should progress first where benefits are clear, and failure modes are manageable and safe, which mirrors early adoption patterns of other clinical decision support tools [50].

Despite this adoption, concern remains pervasive when it comes to errors, bias, privacy, and overreliance. Participants who reported having observed biased outputs in their own use expressed greater concern and stronger support for general safeguards. Respondents more often favored oversight by professional societies and health systems than by model developers or technology companies alone, suggesting a preference for governance based on established clinical norms rather than industry self-regulation. These preferences are consistent with arguments in the existing literature that for AI to be integrated ethically, it must be subject to the same peer review processes and institutional auditing standards as other medical interventions [51,52]. Importantly, our data characterize what health care professionals within this study want to see when it comes to governance, not whether any specific governance approach is effective. Some of the operational directions that align with respondents’ stated preferences, but do not serve as validated recommendations, include tiering use cases by clinical risk, presenting clinician oversight of every LLM-assisted output, requiring task-level testing before deployment, and disclosing LLM involvement in the record. According to the respondents, sustained accountability may benefit from recurring audits with subgroup analyses, incident-reporting pathways, and procurement decisions tied to demonstrated accuracy, calibration, and equity. The comparative effectiveness of these approaches remains an open empirical question that future work should test. This study has several limitations that might affect the generalizability of the findings. All primary outcomes in the study were based on self-reported measures, including perceived knowledge, confidence, concern, and regulatory preferences. These measures reflect subjective assessments rather than externally validated metrics. The survey achieved a response rate of only 1.46%, creating a risk of nonresponse bias.

Respondents who chose to participate may be systematically more engaged with or interested in technology and innovation than the broader population of health care professionals, introducing volunteer or self-selection bias. Consequently, the sample should be considered a self-selective convenience sample rather than a fully representative cross section of health care professionals. Combined with the regional skew toward the Northeastern United States, this sample likely overrepresents tech-forward individuals and institutions and may overestimate LLM awareness, knowledge, adoption, or acceptance relative to the broader health care workforce. Rural health care professionals and those in underresourced communities are underrepresented groups whose perspectives on LLMs may diverge from those predominantly captured in this study. Additionally, self-reported exposure and knowledge are vulnerable to social desirability, recall, and recency effects. As such, the findings should be interpreted as exploratory and hypothesis-generating rather than representative of health care professionals broadly. The cross-sectional design precludes our ability to make true causal attributions regarding the drivers of LLM adoption. Finally, the survey instrument was developed de novo by the research team. Survey items were generated through iterative discussion and consensus among investigators. The instrument was not adapted from a previously validated scale, as no established tool for measuring health care professional perceptions of LLMs was available at the time of study design. The instrument has not undergone formal psychometrical evaluation; therefore, its reliability, construct validity, and other measurement properties remain unknown. However, items were reviewed and refined by the research team for clarity, face validity, and content relevance prior to use. Further work should prioritize additional development and validation of this instrument for measuring LLM-specific perceptions in clinical populations.

In summary, while health care professionals in this sample report targeted early use of LLMs, they maintain sustained concern about safety, bias, and insufficient oversight. A measured adoption strategy anchored in risk tiering, human review, transparent disclosure, and continuous auditing may help support efforts to capture near-term benefits while managing foreseeable risks [53]. Because these data represent attitudes of a limited sample of health care professionals at a single point in time, the attitudes reported here should be read as provisional rather than fixed. As model capabilities advance and regulatory frameworks change, current concerns may ease, while additional ones emerge, potentially redrawing the line between tasks regarded as lower and higher risk. Future work should therefore include longitudinal tracking of attitudes and pragmatic evaluations of how these tools affect documentation quality, workload, patient understanding, and empirical testing of the effectiveness of governance approaches.

### Conclusions

In this exploratory survey of a self-selected convenience sample recruited through an innovation-focused health care mailing list, respondents reported adoption of LLMs primarily for lower-risk tasks and expressed concern about LLM safety, bias, and governance. Given the low response rate and recruitment strategy, these findings should not be interpreted as representative of health care professionals as a whole but as preliminary insights from a subgroup likely to have greater interest in digital innovation and AI.

Within the sample, respondents preferred professional organizations over industry for oversight. Respondents also emphasized the importance of careful planning, human supervision, transparent disclosure, and auditing to support the responsible integration of LLMs into health care settings. Participants suggested that broader adoption of LLMs for higher-stakes clinical applications would require governance models in which professional organizations dictate standards for model validation. These findings reflect stakeholder attitudes rather than evidence supporting any specific governance model. Future research using larger, more representative samples is needed to determine whether these attitudes reflect those of the wider health care workforce. Ultimately, successful integration of LLMs into clinical spaces rests on automation that prioritizes health care professionals as the primary decision makers and ensures that striving for efficiency does not compromise patient safety.

## Supplementary material

10.2196/87818 Multimedia Appendix 1 Survey of health care professionals’ attitudes toward large language models in medicine

## Abbreviations

LLM: large language model
STROBE: Strengthening the Reporting of Observational Studies in Epidemiology
