# Supplementary material for: Attitudes Toward Large Language Models in Health Care and Preferences for Their Adoption and Oversight Among Health Care Professionals: Cross-Sectional Survey
Source: J Med Internet Res. 2026 Sep 15;28:e87818. doi: 10.2196/87818 (PMC13576498; doi:10.2196/87818)
Supplement: Supplementary file 1 [file jmir-v28-e87818-s001.pdf]

# Survey of Large Language Models in Medicine

You are invited to take part in a research study (IRB Protocol #2024P000379) exploring healthcare providers' opinions on large language models (LLMs). This research is unfunded, and your participation is entirely voluntary. You are being invited to participate because you are subscribed to the MESH Incubator email listserv.

Completing this survey will automatically enter you into a raffle to win a $45 Amazon gift card. By completing the survey, you consent to the use of your responses for academic research purposes.

About the Study
LLMs, a subset of Artificial Intelligence (AI), leverage advanced deep learning techniques and vast datasets to understand, generate, and predict new content based on patterns in data ChatGPT, one such LLM which is publicly available, has become immensely popular in the past few months, with applications in numerous fields.LLMs are currently being considered for use In healthcare; given the vast amounts of data available, LLMs are being considered for:

Clinical decision support, such as identifying patients at risk for diseases or recommending optimal treatments
Automating administrative tasks, such as chart reviews and coding
Enhancing patient education with personalized materials
Assisting in research analytics

This survey seeks to evaluate knowledge and attitudes toward LLMs in healthcare settings.

Participation
Participation is voluntary and can stop at any time. Deciding not to participate won't affect medical care they receive at Mass General Brigham now or in the future, or any benefits they receive now or have a right to receive. Deciding not to participate will not affect professional standing or evaluations in the program or department.

Eligibility
Please note that individuals aged 65 and older are not eligible to participate.

Confidentiality and Data Security
All data will be stored on a secure server, and risks of confidentiality breaches are minimized. Your de-identified data may be shared with other researchers for academic purposes without additional informed consent.

Contact Information
For questions or concerns about the study, you may contact Dr. Succi at msucci@mgh.harvard.edu. If you wish to speak with someone independent of the research team about your rights as a participant or have any complaints, you can reach the Mass General Brigham IRB at (857) 282-1900.

Thank you for considering participation in this important research study. Your time and insights are greatly appreciated!

---

Which best describes you?

- ◯ Attending Physician
- ◯ Resident or Fellow Physician
- ◯ Nurse Practitioner
- ◯ Physician Assistant (PA)
- ◯ Nurse
- ◯ Medical Student
- ◯ PA Student
- ◯ NP Student
- ◯ Research
- ◯ Other

---

If you answered 'Other' on the previous question, please specify: ______________________________

---

What is your email (optional, only if you want to enter drawing of gift card)? ______________________________

---

What is your age? ______________________________

| | |
|---|---|
| How many years have you been in practice? (If applicable, write the number of years) | ______________________________ |
| What medical specialty do you practice (if applicable)? | ◯ Anesthesia<br>◯ Internal Medicine<br>◯ General Surgery<br>◯ OB-GYN<br>◯ Radiology<br>◯ Pediatrics<br>◯ Psychiatry<br>◯ Neurosurgery<br>◯ Cardiology<br>◯ Nephrology<br>◯ Dermatology<br>◯ Infectious Diseases<br>◯ Orthopedic Surgery<br>◯ Cardiothoracic Surgery<br>◯ Plastic Surgery<br>◯ GI<br>◯ Endocrinology<br>◯ Hematology/Oncology<br>◯ Emergency Medicine<br>◯ Urology<br>◯ Other |
| If you answered 'Other' on the previous question, please specify: | ______________________________ |
| Which region of the USA do you practice/work in? | ◯ Northeast<br>◯ Midwest<br>◯ Southwest<br>◯ Southeast<br>◯ West |
| Have you used or considered using large language models like ChatGPT in your medical practice? | ◯ Yes<br>◯ No |
| If yes, what kinds of applications have you considered or tried? | _____________________________________ |
| How knowledgeable do you consider yourself when it comes to using ChatGPT or other LLMs in medicine? | ◯ Not at all knowledgeable<br>◯ Slightly knowledgeable<br>◯ Moderately knowledgeable<br>◯ Very knowledgeable<br>◯ Extremely knowledgeable |
| In what areas of medical practice do you think LLMs would be most useful? (select all that apply) | ☐ Clinical decision support (diagnosing and/or treating patients)<br>☐ Answering patient questions<br>☐ Researching medical literature<br>☐ Monitoring patient health<br>☐ Insurance approvals<br>☐ Billing<br>☐ Other |
| If you answered 'Other' on the previous question, please specify: | ______________________________ |

| | |
|---|---|
| How effective do you think Large Language Models like ChatGPT will be in improving efficiency in medical practice? | ◯ Not effective at all<br>◯ Slightly effective<br>◯ Moderately effective<br>◯ Very effective<br>◯ Extremely effective<br>◯ Other |
| If you answered 'Other' on the previous question, please specify: | __________________________________ |
| In your opinion, what are the biggest concerns associated with using Large Language Models in medicine? (select all that apply) | ☐ Data security and privacy<br>☐ Bias in the data and algorithms<br>☐ Lack of transparency in decision-making<br>☐ Overreliance on technology<br>☐ Job loss for healthcare professionals<br>☐ Potential for errors in decision-making<br>☐ Lack of human interaction with patients<br>☐ No concerns<br>☐ Other |
| If you answered 'Other' on the previous question, please specify: | __________________________________ |
| In what ways do you think Large Language Models could perpetuate injustice in healthcare delivery? (select all that apply) | ☐ Bias in decision-making<br>☐ Limited access to healthcare for certain groups<br>☐ Privacy and security concerns<br>☐ Reinforcing existing power dynamics in healthcare<br>☐ Other |
| If you answered 'Other' on the previous question, please specify: | __________________________________ |
| How concerned are you about bias in the development of Large Language Models for use in healthcare? | ◯ Not concerned at all<br>◯ Slightly concerned<br>◯ Moderately concerned<br>◯ Very concerned<br>◯ Extremely concerned |
| In your opinion, what are the potential consequences of bias in Large Language Models for use in healthcare? (select all that apply) | ☐ Incorrect or inaccurate diagnoses<br>☐ Misinformation for patients<br>☐ Disparities in healthcare outcomes for certain groups<br>☐ Distrust in the medical profession<br>☐ Other |
| If you answered 'Other' on the previous question, please specify: | __________________________________ |
| In your experience, have you encountered instances where Large Language Models, like ChatGPT, exhibited biases when applied in healthcare settings? (i.e. situations where these models provided responses that favored certain demographics, underrepresented minority groups, or perpetuated stereotypes in medical advice, diagnosis, or health-related recommendations) | ◯ Yes<br>◯ No<br>◯ Unsure |

| | |
|---|---|
| Do you believe healthcare organizations should be required to disclose any potential biases associated with their use of Large Language Models? (i.e. informing patients or users if a model's training data may lead to inaccuracies or unequal treatment across different demographic groups, such as gender, race, or socioeconomic status) | ◯ Yes<br>◯ No<br>◯ Unsure |
| If you have used an LLM, what was your experience like? (select all that apply) | ◯ Very positive<br>◯ Somewhat positive<br>◯ Neutral<br>◯ Somewhat negative<br>◯ Very negative<br>◯ N/A - have not used an LLM |
| Who do you think should be responsible for regulating the application of Large Language Models in healthcare? | ☐ Government agencies<br>☐ Professional medical associations<br>☐ Healthcare organizations and hospitals<br>☐ Technology companies<br>☐ Other |
| If you answered 'Other' on the previous question, please specify: | __________________________________ |
| How should the use of Large Language Models in healthcare be regulated? (select all that apply) | ☐ Guidelines and standards developed by professional organizations<br>☐ Regulations enforced by government agencies<br>☐ Self-regulation by technology companies<br>☐ Other |
| If you answered 'Other' on the previous question, please specify: | __________________________________ |
| Do you think that healthcare providers should receive specialized training on the use of Large Language Models in healthcare before being allowed to use it on patients? | ◯ Yes<br>◯ No<br>◯ Unsure |
| How confident are you that current regulatory efforts are sufficient to address the potential risks of Large Language Models in healthcare? | ◯ Not at all confident<br>◯ Slightly confident<br>◯ Moderately confident<br>◯ Very confident<br>◯ Extremely confident |
| How can healthcare providers prevent the perpetuation of injustice through the use of Large Language Models? (select all that apply) | ☐ Conducting regular audits of AI systems for bias<br>☐ Ensuring diverse representation in the development and deployment of AI systems<br>☐ Monitoring and addressing disparities in access to healthcare<br>☐ Encouraging patient education and involvement in AI-assisted decision-making<br>☐ Other |
| If you answered 'Other' on the previous question, please specify: | __________________________________ |

| | |
|---|---|
| In your opinion, what steps can be taken to address the potential for Large Language Models to perpetuate injustice in healthcare delivery? (select all that apply) | ☐ Development and enforcement of ethical guidelines for AI use in healthcare<br>☐ Greater transparency and accountability in the development and deployment of AI systems<br>☐ Increased education and awareness of AI's potential for perpetuating injustice<br>☐ Collaboration between healthcare providers, patients, and community organizations to address healthcare disparities<br>☐ Other |
| If you answered 'Other' on the previous question, please specify: | __________________________________ |
| What are your general opinions of using Large Language Models for healthcare? | __________________________________________ |
